# Relative Abundance of Ammonia Oxidizing Archaea and Bacteria Influences Soil Nitrification Responses to Temperature

**DOI:** 10.3390/microorganisms7110526

**Published:** 2019-11-04

**Authors:** Hussnain Mukhtar, Yu-Pin Lin, Chiao-Ming Lin, Yann-Rong Lin

**Affiliations:** 1 Department of Bioenvironmental Systems Engineering, National Taiwan University, Taipei 10617, Taiwan; agricultureenvironment33@gmail.com (H.M.); chiaominglin@ntu.edu.tw (C.-M.L.); 2 Department of Agronomy, National Taiwan University, Taipei 10617, Taiwan; ylin@ntu.edu.tw

**Keywords:** Ammonia oxidizers, MMRT, nitrification, temperature, soil

## Abstract

Ammonia oxidizing archaea (AOA) and bacteria (AOB) are thought to contribute differently to soil nitrification, yet the extent to which their relative abundances influence the temperature response of nitrification is poorly understood. Here, we investigated the impact of different AOA to AOB ratios on soil nitrification potential (NP) across a temperature gradient from 4 °C to 40 °C in twenty different organic and inorganic fertilized soils. The temperature responses of different relative abundance of ammonia oxidizers for nitrification were modeled using square rate theory (SQRT) and macromolecular rate theory (MMRT) models. We found that the proportional nitrification rates at different temperatures varied among AOA to AOB ratios. Predicted by both models, an optimum temperature (T_opt_) for nitrification in AOA dominated soils was significantly higher than for soils where AOA and AOB abundances are within the same order of magnitude. Moreover, the change in heat capacity ($\Delta C_{P}^{‡}$) associated with the temperature dependence of nitrification was positively correlated with T_opt_ and significantly varied among the AOA to AOB ratios. The temperature ranges for NP decreased with increasing AOA abundance for both organic and inorganic fertilized soils. These results challenge the widely accepted approach of comparing NP rates in different soils at a fixed temperature. We conclude that a shift in AOA to AOB ratio in soils exhibits distinguished temperature-dependent characteristics that have an important impact on nitrification responses across the temperature gradient. The proposed approach benefits the accurate discernment of the true contribution of fertilized soils to nitrification for improvement of nitrogen management.

## 1. Introduction

Ammonia oxidation, the rate limiting step of the nitrification process, is carried out by ammonia oxidizing archaea (AOA) and ammonia oxidizing bacteria (AOB) through a key enzyme known as ammonia monooxygenase (AMO), and plays a crucial part in the nitrogen (N) biogeochemical cycle [1,2,3]. Both the AOA and AOB co-exist in diverse soils, and their relative abundance vary from fractional to several orders of magnitude depending upon factors such as soil pH, organic matter, N availability, and carbon to nitrogen ratio [3,4,5,6]. Similarly, the relative contributions of AOA and AOB to soil nitrification may be influenced by many factors, of which temperature is considered to be one of the major factors [7,8,9,10,11,12]. There is direct and indirect evidence that nitrification in soil is dominated by AOB activity at low temperatures, while AOA-based nitrification is dominant at high temperature. For instance, the optimum temperature for the growth of pure cultures of AOB (i.e., *Nitrosospira sp., Nitrosomonas sp.*), isolated from terrestrial and marine environments, is typically ≤ 30 °C [13,14]. In contrast, many studies indicated that the soil-isolated AOA strains (i.e., *Nitrososphaera viennensis, Nitrosocosmicus franklandus (C13), Nitrosotalea sp. Nd2)* showed optimum growth at 35–40 °C [11,15,16]; with few exceptions for the acidophilic Thaumarchaeota strain (*Candidatus Nitrosotalea devanaterra*) [17] and hyperthermophilic strain (*Candidatus Nitrosocaldus yellowstonii*) [18], for which optimum growth occurs at 25 °C and 74 °C, respectively. These observations were further supported by field studies, revealing that AOA and AOB are adapted to take advantage of different temperature ranges for the nitrification process. For instance, Taylor et al. (2012) observed and indicated that the abundance of AOA and their activity increased when soil temperatures reached 30–35 °C, whereas Tourna et al. (2008) reported that in UK agricultural soil, no variation in the AOB community composition was observed when soil was incubated at 25 °C and 30 °C. 

Although AOA and AOB both respond differently to various temperature conditions that have been studied individually, the soil nitrification’s response to temperature in the presence of different relative abundances of ammonia oxidizers remains a major challenge. For instance, previous studies demonstrated that when the abundance of AOA *amoA* in soil was higher than AOB, relatively low nitrification potential rates were observed compared to soils with lower AOA to AOB ratios at 25 °C [6,19,20,21,22], where the studies from Sweden and Oregon forest soils demonstrated a positive correlation for nitrification rate with AOA, but not AOB, when soil was incubated at 25 °C [23,24]. On the other hand, AOA abundance outnumbered the AOB in arctic soils and showed a significant potential nitrification rate at 15 °C, even when AOB *amo*A numbers were below detection [25]. Taylor et al. (2010) showed that the NP rates in Oregon soil, incubated between 22 °C and 40 °C, were considerably higher at 40 °C, versus 22 °C, when the abundance of AOA *amoA* was two orders of magnitude higher than AOB *amoA.* However, there was no considerable difference between nitrification rates at 30 °C and 40 °C for soil exhibiting higher AOA to AOB ratio compared to soils where AOA and AOB abundance was within the same order of magnitude [26]. Although our knowledge on nitrification response to temperature is expanding, we still have an inadequate understanding on nitrification response to temperature in the presence of different relative abundances of ammonia oxidizers. Previous investigations have typically included the influence of different AOA to AOB ratios at certain temperatures such as 25 °C [6,19,20,21,24]; or studying a narrow temperature range which may not adequately capture the emergent behaviors for nitrification arising from the cumulative responses of both AOA and AOB in soils. 

The change in relative abundance of ammonia oxidizers may also influence the cardinal temperatures for nitrification (i.e., optimum temperature (T_opt_), minimum temperature (T_min_), and maximum temperature (T_max_)), since both AOA and AOB have distinct thermodynamic characteristics for ammonia oxidation process [9,10,26]. For instance, previous studies on temperature sensitivity of AOA and AOB reported that the T_opt_ and T_max_ for AOB-supported nitrification potential (NP) was considerably lower than AOA [9]. Furthermore, Duan et al. (2018) found that AOA exhibits a wider temperature range and lower relative temperature sensitivity for nitrification compared to AOB in greenhouse vegetable soils. Although these findings reveal the potential for different AOA to AOB ratios to mediate responses of nitrification fluxes to temperature variability, there still exists a significant knowledge gap about how the temperature sensitivity of soil nitrification is influenced by different AOA to AOB ratios. Therefore, the purposes of this study are (1) to assess the extent to which nitrification responses to temperature are influenced by different relative abundances of AOA and AOB across a temperature gradient (4 °C to 40 °C); and (2) to evaluate relationships between thermodynamic parameters (i.e., cardinal temperatures) and relative abundance of ammonia oxidizers for the soil nitrification process. We hypothesize that proportional nitrification in soil with relatively higher AOA to AOB ratios will be low at temperatures ≤ 25 °C compared to 40 °C. Accordingly, cardinal temperatures (T_min_, T_max_, and T_opt_) associated with nitrification, will increase with increasing AOA to AOB ratios in soil and vice versa. In the presence of different relative abundances of AOA and AOB, the measurement of temperature sensitivity of soil nitrification will help to identify changes in nitrification responses over temperature, in soils that experience a cumulative ammonia oxidizer response.

## 2. Materials and Methods 

To test the above hypothesis, we firstly determined the AOA to AOB ratios for twenty different organic and inorganic fertilized soils and then measured the nitrification potential (NP) rates over a temperature range (4 °C to 40 °C), as the NO_2_ accumulation rate over time. To further quantify the differences in temperature sensitivities of soil NP rates associated with different levels of relative abundances of ammonia oxidizers (i.e., to identify an optimum temperature), we used square rate theory (SQRT) and macromolecular rate theory (MMRT), both of which have been used to determine the temperature sensitivities of enzyme and microbial activities, including soil nitrification processes [9,10].

### 2.1. Study Area and Data Description

Twenty field samples, representing organic (S1–S10) and inorganic (N1–N10) farms covering a range of soil properties and land cover (fallow and cropped soils), were collected from fields located in Miaoli County, Taiwan (24°22′ N, 120°42′ E) (Table S1). The plots were selected through a preliminary examination of archaeal and bacterial abundances and AOA to AOB ratios in soil (as described in Section 2.3). A total of seven sites were selected and screened, resulting in four sites which showed the highest variation for AOA to AOB ratios among plots (Table S1). Plots S1 to S10 were selected from a 1.43 ha organic farm, whereas plots N1 to N10 were selected from three different farms that received chemical fertilizer treatment. Both organic and inorganic farms contain cropped and fallowed soils. We selected these soils because the variation in soil management practices, nitrogen application and cropping pattern are considered to be important factors that result in different relative abundance of AOA and AOB [8,27] and so nitrification potential. The physicochemical characteristics, total area, and crop type for organic and inorganic plots are shown in the supplementary material (Table S1). In order to determine the soil nitrification potential (NP) response, three to five soil samples from each plot were collected randomly at depths of approximately 15 cm for each replicate. A composite sampling technique was used to represent the sampled field, and sieved through the mesh to get particle size < 3 mm. The samples were stored at 4 °C and −20 °C for physicochemical and molecular analysis, respectively. The soil NP response to temperature was determined by exposing the (autotrophic) ammonium-oxidizer to ammonium sulfate in soil slurry buffered at pH 7.2, and incubating the slurry in a range of temperatures (4 °C to 40 °C). Twelve and half grams (12.5 g) of moist soil was used for each replicate (*n* = 3). The soil slurry nitrite and nitrate concentrations were monitored for 3 days at low incubation temperatures (4 °C and 17 °C) and 1 day at high incubation temperatures (25 °C, 32 °C, and 40 °C) as described by Mukhtar et al. (2019). Sodium chlorate (NaClO_3_, 7.4 ± 0.1 mmol/L) was used to inhibit the oxidation of the nitrite, which is carried out by nitrite-oxidizing bacterial activity in the slurry. The rates of accumulation of nitrite over time in the soil slurries were considered to be the NP rates, and were measured using linear regression between concentrations and time. A detailed procedure for the soil NP method is reported by Mukhtar et al. (2019). In order to compare the nitrification response carried out by different relative abundances of ammonia oxidizers at different temperatures, the proportional nitrification potential (%) was calculated with the following formula:

$$\mathrm{Proportional}\;\mathrm{NP}\;\left(\%\right)=\frac{\mathrm{Nitrification}\;\mathrm{at}\;a\;\mathrm{specific}\;\mathrm{temperature}}{\mathrm{Cumulative}\;\mathrm{nitrification}\;\mathrm{across}\;\mathrm{the}\;\mathrm{temperature}\;\mathrm{gradient}}\times 100$$

### 2.2. Model Construction

Nitrification potential response was modeled with a SQRT model as a function of temperature. SQRT has been used to model and simulate bacterial growth [28,29], soil respiration [30], and NP response to temperature [9,31]. NP rates were simulated across the entire temperature range based on the following function proposed by Ratkowsky et al. (1983) [28] for bacterial growth in response to temperature [28]:

(1) $$\sqrt{Bacterial\;growth}=a\left(T-T_{min}\right)\left(1-e^{b\left(T-T_{max}\right)}\right)$$

where T_min_ and T_max_ represent the minimum and maximum temperature, respectively, at which nitrification potential may occur. T_min_ was used as a theoretical constant to describe the x-intercept. *a* and *b* are parameters for a nitrification potential rate below the optimum temperature and a nitrification decrease rate above the optimum temperature (T_opt_), respectively. T_opt_ was calculated by taking the first derivate of Equation (1). Since the variable *T* can’t be solved explicitly for Equation (2) (first derivate of Equation (1)), the R-function ‘uniroot’ was used to determine the *T* at which the solution of Equation (2) was approximately equal to zero (<0.00001), and estimated temperature (*T*) was considered as T_opt_ for NP activity.

(2) $$-a\;\left((b\left(T-T_{min}\right)+1\right)e^{\left(b\left(T-T_{max}\right)\right)}-1)=0$$

NP response was also modeled with a MMRT model as a function of temperature. MMRT has been used to model and simulate microbial activities and nitrification rates, which are measured as per the following mathematical expression [9]:

(3) $$\ln\left(k\right)=\ln(\frac{K_{B\;}T}{h})-\;\frac{\Delta H_{To}^{‡}+\;\Delta C_{P}^{‡}\;\left(T-T_{O}\right)\;}{RT}+\frac{\Delta S_{To}^{‡}+\Delta C_{P}^{‡}\;\left(\ln\left(T\right)-\ln(T_{O})\right)\;}{R}$$

where k is the rate constant, T_o_ (298.15 K, 25 °C) is the reference initial temperature for the fitting process [32], R is the universal gas constant (8.314 JK^−1^mol^−1^), h is Planck’s constant, and k_B_ is Boltzmann’s constant. $\Delta H_{To}^{‡}$, $\Delta S_{To}^{‡}$, and $\Delta C_{P}^{‡}$ represent the change of enthalpy, change of entropy, and change in heat capacity related to temperature-dependent nitrite accumulation rates that are attributed to ammonia oxidizers, respectively. The activation enthalpy follows a linear relation with temperature variation, while entropy varies with the natural log of the temperature [33]. Yet, the change in heat capacity is sensitive to enzyme flexibility [34] and depends upon enzyme type and microbe-enzyme association [35]. T_opt_ for the MMRT model was calculated by using the following equation [32]:

(4) $$T_{opt}=\;\frac{\Delta H_{To}^{‡}-\;\Delta C_{P}^{‡}T_{0}\;}{-\Delta C_{P}^{‡}-R}$$

### 2.3. DNA Extraction and Quantification of Ammonia Oxidizer Populations

DNA was extracted from 0.5 g dried soil samples with a ‘FastDNA SPIN kit’ for soil (MP Biomedicals, Solo, OH, USA). DNA extracts were then quantified using a ‘NanoDrop’ spectrophotometer (NanoDrop 1000, Thermo Fisher Scientific, DE, USA). The extracted DNA was stored at −20 °C for further analysis. For quantification of AOA and AOB by real-time polymerase chain reaction (PCR), amplification was performed in 20 μL reaction mixtures using ‘iQTM SYBR GREEN Supermix’ and a ‘CFX CONNECT Real-Time PCR Detection System’ (Bio-Rad laboratories, Hercules, CA, USA). In this study we used 16S rRNA to determine the AOA and AOB population in soil due to its smaller variation in copy numbers of AOB—16S rRNA per cell [36,37,38,39,40], (i.e., AOB dominant species in soil such as *Nitrosospira sp.* usually exhibit one copy compared to the AOB-*amo*A gene which exhibits 2 to 3 copies). Primers 189fC (GGAGGAAAGTAGGGGATCG) and RT1r (CGTCCTCTCAGACCAACTACTG) were used to quantify AOB 16S ribosomal RNA gene abundances [41]. However, primers AP422F (GTCTAAAGGGTCTGTAGCCG) and AP599R (TTCTGGTGAGACGCCTTCG) were designed in this experiment to quantify AOA 16S ribosomal RNA gene abundances, while the specificity of primers was validated by AOA 16S rRNA sequence cloned plasmids (see supplementary material). Each 20 μL reaction volume contained 20 ng soil DNA extract, both 100 μM forward and reverse primers, 10 μL 2-fold Supermix buffer and 5.5 μL double distilled water (DDW). The amplification conditions were as follows: 2 min at 50 °C and 5 min at 95 °C, followed by 40 cycles of 15 s at 95 °C, 40 s at 60 °C, and 30 s at 72 °C, then 10 min at 72 °C. 

### 2.4. Statistical Analysis

Nash–Sutcliffe coefficient (NSE) was used as a goodness-of-fit criterion and likelihood measure, defined as follows [42]:

(5) $$L\;\left(\theta_{i}|Y\right)=\left(1-\sigma_{i}{}^{2}/\sigma_{\mathrm{obs}}{}^{2}\right)$$

where $L\;\left(\theta_{i}|Y\right)$ is the measure of likelihood for the model simulation on a set of observations, and $\sigma_{i}{}^{2}$ and $\sigma_{\mathrm{obs}}{}^{2}$ denote the associated error variance and observed variance, respectively. Unknown parameters (*a*, *b*, T_min_, T_max_, $\Delta H_{To}^{‡}$, $\Delta S_{To,}^{‡}$ and $\Delta C_{P}^{‡}$) were fitted by a nonlinear least squares estimation method (nls) and Markov Chain Monte Carlo (MCMC) algorithm in the R programming language. That is, the R-function ‘nls’ was used to obtain approximate values for unknown parameters, then an MCMC algorithm was used to further approximate values until an NSE coefficient value greater than 0.6 was reached. 

Analysis of variance (ANOVA) with the Tukey-Kramer adjustment was used to test whether organic and inorganic farms including cropped and non-cropped soils significantly influence the ammonia oxidizers abundances, their ratios, and NP activities at different temperatures. In case of AOA to AOB ratios, data were log transformed (base of 10). Multiple regression analysis was performed to determine correlation between estimated thermodynamic parameters and relative abundance of ammonia oxidizers, and intra-parameter correlations. A *t*-test (t distribution) was used to evaluate significance for Pearson correlation coefficients, a determinate for regression analysis. All statistical analysis was performed using R programming language. Complete summaries of the statistical analysis are contained in supplementary material.

## 3. Results

### 3.1. Effect of AOA to AOB Ratios on Nitrification Potential over Temperature Gradient 

**Figure 1:**
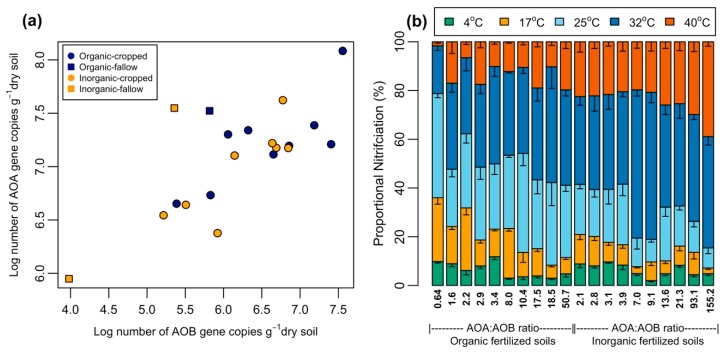
The variation (**a**) in ammonia oxidizing archaea (AOA) and ammonia oxidizing bacteria (AOB) abundances (qAOA and qAOB, respectively) among organic and inorganic farm and influence of AOA to AOB ratios (**b**) on soil proportional nitrification across a temperature range of 4 to 40 °C. Error bars represent the standard error (*n* = 3) for nitrification potential at each temperature.

The number of AOB genes was in the range of 0.95 × 10^4^ − 36.06 × 10^6^ gene copies/g of dry soil, whereas the AOA genes varied between 8.9 × 10^5^ − 12.13 × 10^7^ gene copies/g of dry soil (Figure 1). The AOA abundance was significantly higher than AOB in the fallow (unfertilized) plots compared with the cropped plots (*p* < 0.05) (Figure 1). Consequently, in both farming systems, the highest AOA to AOB ratio was found in the fallow (unfertilized) plots (Figure 1) where there was no significant difference (*p* > 0.05) in relative abundances of AOA to AOB between cropped plots. The AOA to AOB ratios in fallow plots varied from 50.7 to 155.2, which were significantly higher than the AOA to AOB abundances found in the cropped soils (0.64 to 21.3). Among both farming systems, AOA abundance in inorganic soil was negatively correlated with total organic carbon (TOC), and the carbon to nitrogen ratio (C:N). No significant correlation (*p* > 0.05) was observed between AOA and AOB abundance and soil properties in organic farm samples (Table S3).

The data on NP rates associated with the different AOA to AOB ratios (0.64 to 155.2), over a temperature gradient, demonstrated that the NP rates were significantly higher in soil samples with relatively high AOA to AOB ratios at a high temperature (40 °C), compared to the soil samples where AOA to AOB ratios were within the same order of magnitude. By contrast, at 25 °C, lower proportional NP rates correspond to high AOA to AOB ratios, regardless of soil type (Figure 1). The NP rates in soils with a high AOA to AOB ratio increased significantly (*p* < 0.05) from 4 °C to 32 °C, and then declined. At a relatively lower AOA to AOB ratio, NP rates usually increased to 25 °C, and significantly (*p* < 0.05) decreased at 40 °C. The maximum proportional NP rates were observed at 32 °C (38.7 ± 8.2 %) compared to 25 °C (24.1 ± 9.98%) and 40 °C (18.5 ± 9.2%), with relatively slight variation throughout the soil samples. However, proportional NP rates were considerably higher at 25 °C than those at 32 °C for organic soil samples where AOA to AOB ratio <1.

### 3.2. Variation in Temperature Sensitivity Traits

In order to further understand the thermodynamic differences between different relative abundances of ammonia oxidizers for nitrification, the NP rates in response to temperature was fitted using two complementary models, SQRT and MMRT. Table S2 summarizes the results of the NP simulation over a temperature range (4 °C to 40 °C). The NSE coefficient values of the fitted NP values using the SQRT and MMRT models were 0.82 ± 0.09 and 0.78 ± 0.12, respectively, while the corresponding correlation constant (*r*) values for the fitted NP values from both models were 0.92 ± 0.05 and 0.89 ± 0.06, respectively. The SQRT model simulated the maximum NP rates at 32 °C and the sudden drop in NP rates at 40 °C and thus, showed high NSE and correlation coefficient values for both organic and inorganic soils. However, the MMRT model showed high NSE and correlation constant values for soils that showed relatively less fluctuation in NP rates (smooth curve [43]) at different temperatures. 

**Figure 2:**
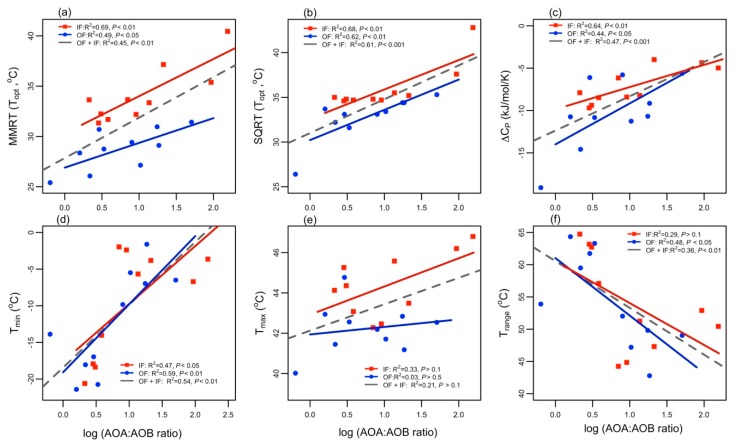
The influence of AOA to AOB ratios on (**a**) mean optimum temperature for nitrification estimated by the macromolecular rate theory (MMRT) model; (**b**) optimum temperature estimated by the square root theory (SQRT)) model; (**c**) change in heat capacity associated with the temperature dependence of nitrification ($\Delta C_{P}^{‡}$); (**d**) theoretical apparent minimum temperature (T_min_) for NP activity; (**e**) maximum temperature (T_max_) for NP activity; and (**f**) temperature range (T_max_–T_min_). “OF” and “IF” represent the soil samples from organic and inorganic fertilized soils, respectively.

When the NP responses to temperature, associated with different AOA to AOB ratios, were compared, several thermodynamic parameters estimated by SQRT and MMRT models were significantly correlated with the AOA to AOB ratios. Our multi-regression analysis revealed that the T_opt_ for NP activity increases with increasing AOA to AOB ratios (Figure 2). However, the T_opt_ was better correlated with the AOA to AOB ratios in organic and inorganic fertilized soil than the correlation between T_opt_ and the AOA to AOB ratios as a whole. This observation was true for estimations of T_opt_ using both SQRT and MMRT models (Figure 2a,b). However, the T_opt_ values estimated by the SQRT model (34.37 ± 2.95), were considerably higher compared to those estimated by the MMRT model (31.42 ± 3.66), regardless of different soil types. In addition, the SQRT model estimated a theoretical apparent minimum temperature of activity (T_min_), which showed positive correlation (R^2^: 0.47 and 0.59, *P*: <0.05 and <0.01 for inorganic and organic fertilized soils) with increasing AOA to AOB ratios (Figure 2). However, the SQRT model’s maximum temperature of activity showed weak correlation with the AOA to AOB ratios and varied slightly among the groups.

**Figure 3:**
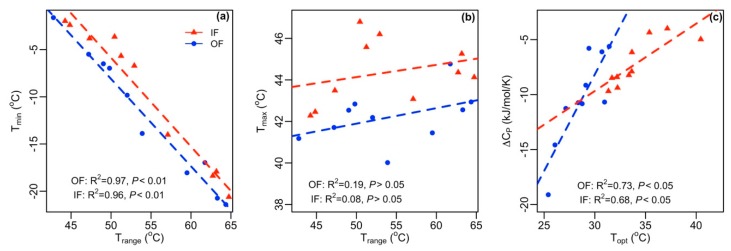
Relationship between temperature ranges and (**a**) minimum temperature for activity; (**b**) maximum temperature for activity; and (**c**) correlation between change in heat capacity associated with the temperature dependence of nitrification ($\Delta C_{P}^{‡}$) and optimum temperature predicted by the MMRT model.

The temperature ranges (T_max_–T_min_) estimated by the SQRT model were highly sensitive to T_min_ (Figure 3), decreased with an increasing AOA to AOB ratio, and were correlated relatively stronger for organic soil. The change in heat capacity associated with the temperature dependence of nitrification $\left(\Delta C_{P}^{‡}\right)$ estimated by the MMRT model, ranged from −4.74 to −19.11, and −2.61 to −11.38 kJ mol^−1^ K^−1^ for organic and inorganic soils, respectively. This supports previously established values for soil nitrification [9,10]. The parameter $\Delta C_{P}^{‡}$ was positively correlated with the MMRT-estimated T_opt_, and increased (less negative) with an increasing AOA to AOB ratio, though it maintained a strong relationship in inorganic soil compared to organic fertilized soil.

## 4. Discussion

### 4.1. Nitrification Responses to Temperature Vary among AOA to AOB Ratios

Our study documents the variations in nitrification responses and associated cardinal temperatures, and explores how they diverge for different relative abundances of ammonia oxidizers across a temperature gradient (4 °C to 40 °C). The results showed that the proportional NP rates increase with increasing AOA to AOB ratio at 40 °C, while an opposite trend was observed when incubation temperatures were <32 °C. This observation was true for both farming systems fertilized regularly with organic and inorganic fertilizers. Although the literature is sparse in this area, the nitrification response has been examined previously for specific temperatures such as 25 °C [6,19,20,21,22], and for relatively narrow temperature ranges [26]. The studies generally found that larger nitrification values correspond to lower AOA to AOB ratios at 25 °C, and to maximum nitrification rates at 30 °C (Taylor et al., 2010) which corroborate our results. This similarity in results is likely related to the influence of distinct and characteristic responses of AOA and AOB across a temperature gradient since soil nitrification is usually carried out by AOA at high temperatures (>35 °C), whereas AOB are more active at temperatures ≤ 30 °C. Thus, soils with lower AOA to AOB ratios experience a relatively higher proportion of AOB nitrification activity at lower temperatures, compared to soils where AOA abundance is dominant (Figure 1). However, the increased nitrification at 32 °C for both organic and inorganic soils may be associated with the cumulative activity of ammonia oxidizers, since both AOA and AOB could be active for nitrification at 32 °C, as observed in previous studies [26]. Differing from the results of previous studies, we found that nitrification rates increased with increasing AOA to AOB ratios in soil at 40 °C; and NP rates were significantly higher at 40 °C than 25 °C for highly AOA dominant soils despite the maximum nitrification at 32 °C. Our results suggest that above 32 °C, increases in temperature may further accentuate AOA-supported nitrification; and proportional NP increase with increasing AOA to AOB ratio in various soils, especially for inorganic fertilized soils which showed high AOA to AOB ratios than organic fertilized soils. These results concur with previous studies who report optimum growth for soil AOA isolates at temperatures >35 °C [11,15,16]. In contrast, a lower nitrification rate at 4 °C, for AOA abundant soils, is evidence that AOB is likely the major player for soil nitrification at low temperatures [44]. Such findings demonstrate a span of multiple temperature-dependent characteristics for nitrification, suggesting that a change in ratio of AOA to AOB in soil results in deferential contribution to soil nitrification over a range of temperature.

Both SQRT and MMRT models yield similar results, indicating that the optimum temperature (T_opt_) for nitrification activity increases with increasing AOA to AOB ratios, supporting our hypothesis. Although it is unknown from the literature if an increase in AOA to AOB ratios will increase T_opt_ for nitrification and vice versa, nitrification response to temperature has been modeled individually for AOA and AOB-based nitrification [9,10], and these results partially corroborate our findings. At the outset of this study, we expected to find that the T_opt_ range for nitrification, in the presence of both AOA and AOB, would lie between those limits that have been previously (and separately) reported for AOA and AOB. However, the range of the T_opt_ observed in this study among different AOA to AOB ratios for nitrification activity (estimated T_opt_ values 25.4–40.4 °C and 26.4–42.8 °C for MMRT and SQRT models, respectively) was similar to or greater than those using the octyne resistance method to determine temperature niche separation for AOA and AOB [9,10]. Although it is not well established if octyne is sensitive to temperature [9], our results raised the possibility that some AOB strains may be partially sensitive to octyne, which in turn may underestimate the potential functions of AOA and AOB for nitrification. 

Although the variation in the T_opt_ parameter modeled by the MMRT and SQRT models is the result of multiple responses of AOA and AOB for nitrification, it is intriguing to consider T_opt_ in the context of changes in heat capacity ($\Delta C_{P}^{‡}$) when explaining the conformational states of AMO. Among temperature sensitivity parameters, $\Delta C_{P}^{‡}$ was positively correlated (*p* < 0.01) with T_opt_. These observations are in line with previous studies, which hypothesized that the large negative $\Delta C_{P}^{‡}$ values usually relate to a lower T_opt_ for enzyme activities across a temperature gradient [9,33,34]. The $\Delta C_{P}^{‡}$ has been estimated previously for AOA and AOB separately. In general, these studies found relatively less negative $\Delta C_{P}^{‡}$ values for AOA when compared with AOB. This implies that the AOA AMO may be more rigid and constrained than the AOB AMO [9,45]. Thus, the decline in $\Delta C_{P}^{‡}$ (large negative $\Delta C_{P}^{‡}$) value with decreasing AOA to AOB ratios indicates that soils, which are approximately equally abundant with AOA and AOB, may provide a broader range of AMO flexibility for ammonia oxidation processes. By contrast, as the AOA to AOB ratio increases, ammonia oxidizers exhibit a narrow range of AMO flexibility. Thus, $\Delta C_{P}^{‡}$ increased (less negative) and significantly high T_opt_ were observed when compared to soil where the AOA to AOB ratio was within the same order of magnitude. It is interesting to note, however, that the MMRT model estimated a smaller T_opt_ value compared to the SQRT model, which may be the result of a difference in model performance. Similar to the results from the previous studies [9,43], the SQRT model fitted the temperature sensitivity of NP rates better than the MMRT model. However, if the curvature of the NP response curve was relatively smooth over the temperature variation, then there was little or no difference between fits based on SQRT and MMRT models (Table S2). The MMRT model fitted the NP response to temperature as an exponential function so that the sudden drop in an NP rate at 40 °C was not simulated; the SQRT model, however, fitted temperature sensitivity with high accuracy at lower and higher temperatures because the second term in Equation (1) $\left(1-e^{b\left(T-T_{max}\right)}\right)$ gained importance as temperatures approached T_opt_, though the opposite was true as temperatures fell below T_opt_ [29]. These factors are likely contributing to differences in optimum temperatures estimated by both SQRT and MMRT models.

The AOA abundant soil provides a relatively narrow temperature range for nitrification despite the weak, or nonexistent, relationship between T_max_ and AOA to AOB ratios. A wider temperature range for nitrification for lower AOA to AOB ratios may presumably be caused by greater variability in T_min_, which showed high correlation with temperature ranges compared to a SQRT-model-predicted T_max_. These results agree with Duan et al. (2018) who found differences in thermodynamic responses of AOA and AOB; and temperature ranges were more influenced by parameter T_min_ than T_max_. When looking deeper into the characteristics of the nitrification response curve, we found some support for this behavior. The parameter T_max_ represents the maximum temperature below which substrate turnover and *de novo* protein synthesis occur to resume the activity [33,46]. Therefore, T_max_ in diverse soils is less likely to be influenced by different AOA to AOB ratios due to the lack of cumulative contribution of AOA and AOB in shaping the maximum allowable temperature for the nitrification process. In contrast, such increased variability in T_min_ may be associated with AOB, which are more active for nitrification at temperatures ≤ 30 °C [9,13,14]. Therefore, a small variation in the relative abundance of AOA and AOB may have relatively higher potential to show greater variation in proportional nitrification at a low temperature (<30 °C), when the abundances of AOA and AOB are within the same order of magnitude. By contrast, as the AOA to AOB ratio increases up to a threshold, NP response over a temperature gradient may be dominated by the characteristic responses of AOA; while above this threshold, response variations result with changes in AOA abundance that theoretically would be smaller than the variation caused by cumulative response of both AOA and AOB. Subsequently, T_min_ decreased with increasing AOA to AOB ratios and showed relatively narrow temperature ranges for AOA-dominated soils. These results also help to clarify interactions among cardinal temperature (T_min_ and T_max_) and ammonia oxidizer characteristics in AOA-dominated soils that give rise to temperature ranges not apparent when investigating the effects of T_max_ separately.

Our results indicated that the correlation between thermodynamic parameters and relative abundances of ammonia oxidizers was considerably different among organic and inorganic fertilized soils. This discrepancy could be associated with the magnitude of AOA to AOB ratios for which variation was significantly higher for inorganic fertilized plots compared to plots in organic farms. Although, the maximum AOA to AOB ratio was observed in fallow (unfertilized) plots for both farming systems, only AOA abundance in inorganic farm samples showed negative correlation with soil properties, such as total organic carbon and carbon to nitrogen ratio. The observed higher AOA to AOB ratio in fallow (unfertilized) than in cropped (fertilized) soil, is consistent with existing studies that found an increase in AOA abundance in unfertilized soils [8] where AOB abundance was strongly stimulated after application of ammonium fertilizer [27]. However, a decrease in total organic carbon in our soils may further accentuate the variability in AOA, and associated AOA to AOB ratios, in fallow plots of inorganic farm samples. Consequently, a range of AOA to AOB ratios in organic farm samples, that are relatively narrower than that in inorganic farm samples, showed a small variation in nitrification response, and so, also in thermodynamic parameters and their correlation with relative abundances of ammonia oxidizers. However, it is of some importance to consider that the thermodynamic parameters estimated by the SQRT and MMRT models were considerably different for numerically similar AOA to AOB ratios from the organic farms than the inorganic fertilized farms. Such results might be associated with wider physiological diversity (sub groups) of AOA and AOB, which exhibit varying size, sensitivity to ammonia concentration, and specific cell activity [11,16] with different nitrification characteristics within the AOA or AOB group. Although, in this study, all soil samples were collected under the same environmental conditions, the difference in management practices between organic and inorganic farms (i.e., fertilizer type) may influence the AOA and AOB composition in the two farming systems [23,47] and so exhibited nitrification responses to temperature may differ for similar AOA to AOB ratios in soils. However, it remains to be seen if changes in AOA to AOB ratios from other soil environments will yield similar results, and how mixed soil populations that are dominated with different ammonia oxidizers clusters, respond to temperature changes for nitrification process. 

### 4.2. Implications

The results presented here support the assertion that various AOA to AOB ratios contribute differently to soil nitrification across a temperature gradient as discussed in the above section. Variability in NP responses to temperature was clearly organized according to relative abundances of AOA and AOB in soil. Although we readily concede that the magnitude of the variability might vary across different ecosystems due to wider diversity of AOA and AOB, this study has highlighted several points which are important in order to understand the temperature response of soil nitrification in the presence different relative abundances of ammonia oxidizers. For instance, our study challenges the general framework adopted in recent studies to compare nitrification in different soils at a fixed temperature. We demonstrated that measuring NP rates at a fixed temperature (i.e., 25 °C) in soils exhibiting different AOA to AOB ratios represent the proportion of activity dominated by a specific group of ammonia oxidizers (i.e., AOB). Therefore, overlooking the temperature dependence of the nitrification responses associate with different AOA to AOB ratios, may lead to an underestimation or overestimation of NP rates, and further to profound impacts on the large-scale nitrogen balance in soils. An appropriate temperature, based on the relative abundance of AOA and AOB, is essential to accurately discern the true contribution of ammonia oxidizers, and to differentiate the nitrification potential in fertilized soils. 

Additionally, soil nitrification responses to temperature can be summarized in terms of three cardinal temperatures, namely the base or minimum (T_min_) at which ammonia oxidizers are able to resume activity, the optimum (T_opt_) where the highest rate of activity shows, and maximum (T_max_) temperatures above which nitrification activity no longer occurs. Our estimations of cardinal temperatures with the SQRT and MMRT models for nitrification suggest that optimum temperature for nitrification will be considerably high in AOA dominant soils compared to soils where relative abundance of AOA and AOB will be within the same order of magnitude. On the other hand, AOA dominated soils will result in narrow temperature ranges for nitrification. Representing this type of behavior in our findings may help to optimize the N fertilizer applications in soils exhibiting different AOA to AOB ratios, as well as crop production.

## 5. Conclusions

We have measured the nitrification response to temperature associated with different relative abundances of ammonia oxidizers across a temperature gradient from 4 °C to 40 °C. By modeling the temperature response with SQRT and MMRT models, we isolated the important characteristics (T_min_, T_max_, T_opt_, and $\;\Delta C_{P}^{‡}$) of ammonia oxidizers for the nitrification process. We found that the temperature sensitivity of soil nitrification significantly varies among different relative abundance of ammonia oxidizers, even though the AOA to AOB ratio varies at different magnitudes among farming systems. Our results explain what has previously been observed, and also provides insight into how different AOA to AOB ratios in soil contribute differentially to nitrification. This indirectly supports the assertion that AOA and AOB contribute differently to soil nitrification across a temperature gradient. However, these results contradict the generally-adopted framework of prior studies, that focus on measuring soil nitrification responses at fixed temperatures among various soils exhibiting different relative abundance of AOA and AOB. We suggest that measuring soil NP rates at a fixed temperature (i.e., 25 °C) represents the proportion of activity dominated by a specific group of ammonia oxidizers. Appropriate temperatures based on the relative abundance of AOA and AOB is therefore essential to accurately discern the true nitrification potential of various soils. Future research is required to understand the extent to which the various dominant clusters of AOA and AOB, and their relative abundances influence the temperature sensitivity of soil nitrification.

## Supplementary Materials

The following are available online at https://www.mdpi.com/2076-2607/7/11/526/s1, Table S1: Edaphic properties of selected organic (OF-1 to OF-10) and inorganic (IF-1 to IF-10) fertilized soil, Table S2: Thermodynamic parameter estimation and accuracy of SQRT and MMRT models fit to measured NP rates. O-F1 to OF-10 and IF-1 to IF-10 represent organic and inorganic fertilized soils. Note; a variation of 0.015 for likelihood functions (NSE and *r*) may be possible while replicating these results since sensitive parameters such as Tmax and *a* are rounded up to two to three decimal digits, respectively, from more than five decimal digits, Table S3: Correlation (Pearson correlation coefficients) between soil properties and AOA to AOB ratios. Values in parentheses indicate significance values (*P*) determined by using t distribution.
