# Supplementary material for: Relative Abundance of Ammonia Oxidizing Archaea and Bacteria Influences Soil Nitrification Responses to Temperature
Source: Microorganisms. 2019 Nov 4;7(11):526. doi: 10.3390/microorganisms7110526 (PMC6920900; doi:10.3390/microorganisms7110526)
Supplement: Supplementary file 1 [file microorganisms-07-00526-s001.pdf]

Contents of this file

Description of AOA primer
Tables S1 to S3

Introduction

Supplementary information contains description of AOA primer (Figure S1) and three tables with basic information on physio-chemical characteristics, total area, and crop type for organic and non-organic soil plots (S1), the accuracy of the unknown parameter estimation results for SQRT and MMRT models (S2) and correlation (Pearson correlation coefficients) between soil properties and AOA to AOB ratios (S3).

Description of AOA primer

For quantification of AOA 16S ribosomal RNA gene abundances, we designed the primer pair of AP422F (GTCTAAAGGGTCTGTAGCCG) and AP599R (TTCTGGTGAGACGCCTTCG) in this experiment. The specificity of primers was confirmed by comparing the PCR products of organic/inorganic soil samples and the plasmids which AOA 16S ribosomal RNA gene of two major genera (*Nitrososphaera* and *Nitrosopumilus*) from soil samples were cloned. The result showed both organic/inorganic soil samples and AOA 16S ribosomal RNA gene cloned plasmids has the same size of PCR products (178bp) by using the primers designed for this experiment (Fig. S1). In addition, the melting curve of qPCR is consistent to ensure the specificity of products.

Supplementary Figures

Supplementary tables

Table S1. Edaphic properties of selected organic (OF-1 to OF-10) and inorganic (IF-1 to IF-10) fertilized soil.

Soil sample	Number of plots	Farm type	Cropping patterns	Total Area (hectare)	pH*	TN (g/kg of dry soil)	TOC (g/kg of dry soil)	C/N ratio
OF-1 to OF-10	10	Site 1: duck-paddy organic farm	Fallow and Paddy	1.43	6.55-7.51	1.16-1.79	11.54 - 20.03	7.75-13.61
IF-1 to IF-6	6	Site 2: Non-organic farm	Fallow-Wheat-paddy	1.02	6.57-7.10	1.43-1.96	6.47 – 10.55	3.74-7.48
IF-7 to IF-8	2	Site 3: Non-organic farm	Paddy	0.28	6.71-6.93	1.27-1.35	5.15 – 6.87	3.81-5.41
IF-9 to IF-10	2	Site 4: Non-organic farm	Fallow-paddy	0.46	6.60-6.78	1.33-1.67	8.37 – 9.08	5.44-6.29

*Soil to water ratio (1:2.5), OF: Soils from organic farm, IF: Soils from inorganic farm, TN: Total nitrogen, TOC: Total organic carbon, C/N: TOC to TN ratio

Table S2. Thermodynamic parameter estimation and accuracy of SQRT and MMRT models fit to measured NP rates. O-F1 to OF-10 and IF-1 to IF-10 represent organic and inorganic fertilized soils. Note; a variation of ±0.015 for likelihood functions (NSE and *r*) may be possible while replicating these results since sensitive parameters such as T_{max} and *a* are rounded up to two to three decimal digits, respectively, from more than five decimal digits.

**Soil sample**	**Square root growth (SQRT)**							**Macromolecular rate theory (MMRT)**					
	a	*b*	T_{min}	T_{max}	T_{opt}	NSE*	*r***	$\Delta H^{\ddagger}_{To}$	$\Delta C^{\ddagger}_{P}$	$\Delta S^{\ddagger}_{To}$	T_{opt}	NSE*	*r***
	------	------	^{O}C	^{O}C	^{O}C	-------	-----	$k.J.mol^{-1}$	$k.J.mol^{-1}.K^{-1}$	$k.J.mol^{-1}$	^{O}C	-------	------
OF-1	0.013	0.304	-18.05	41.45	32.2	0.93	0.97	10.550	-14.58	-0.234	26.07	0.70	0.90
OF-2	0.022	0.138	-13.88	40.02	26.4	0.86	0.92	1.20	-19.11	-0.266	25.41	0.81	0.93
OF-3	0.013	0.231	-20.74	42.56	31.6	0.81	0.90	36.82	-10.83	-0.142	28.75	0.66	0.86
OF-4	0.009	0.315	-21.41	42.94	33.7	0.93	0.97	32.310	-10.74	-0.162	28.36	0.65	0.90
OF-5	0.016	0.412	-1.61	41.18	34.4	0.91	0.97	34.420	-9.15	-0.153)	29.11	0.79	0.90
OF-6	0.007	0.212	-16.97	44.77	33.1	0.84	0.94	32.71	-6.11	-0.161	30.70	0.81	0.91
OF-7	0.015	0.31	-5.49	41.71	33.4	0.79	0.90	20.170	-11.26	-0.201	27.14	0.88	0.94
OF-8	0.016	0.315	-6.97	42.84	34.4	0.96	0.99	59.96	-10.68	-0.067	30.96	0.96	0.99
OF-9	0.019	0.398	-6.5	42.54	35.3	0.89	0.95	34.13	-5.62	-0.145	31.42	0.84	0.92
OF-10	0.02	0.285	-9.83	42.19	33.1	0.95	0.98	23.598	-5.792	-0179	29.42	0.81	0.91
IF-1	0.013	0.251	-17.92	45.26	34.6	0.81	0.91	58.090	-9.69	-0.070)	31.34	0.74	0.92
IF-2	0.012	0.298	-18.37	44.36	34.8	0.73	0.87	64.660	-9.38	0.050	32.24	0.78	0.91
IF-3	0.023	0.315	-3.82	43.49	35.2	0.79	0.89	47.05	-3.99	-0.100	37.15	0.66	0.83
IF-4	0.027	0.340	-2.39	42.46	34.7	0.64	0.84	57490	-8.41	-0.065	32.19	0.57	0.77
IF-5	0.01	0.324	-20.61	44.13	35.0	0.84	0.93	65.490	-7.899	-0.048	33.64	0.69	0.93
IF-6	0.021	0.981	-3.64	46.8	42.8	0.70	0.85	75.04	-4.97	-0.007	40.45	0.81	0.90
IF-7	0.029	0.36	-1.97	42.28	34.8	0.67	0.84	51.13	-6.15	-0.0858	33.66	0.52	0.74
IF-8	0.025	0.239	-5.67	45.58	35.5	0.82	0.92	65.97	-8.23	-0.037	33.36	0.91	0.96
IF-9	0.02	0.316	-6.71	46.2	37.6	0.76	0.87	43.630	-4.35	-0.110	35.38	0.75	0.86
IF-10	0.017	0.349	-14.02	43.08	34.7	0.84	0.93	53.790	-8.49	-0.081	31.69	0.86	0.93

* NSE: Nash-Sutcliffe coefficient

** *r:* Correlation constant<br><br>*a* and *b* : Cofficient for SQRT model<br><br>T_{min}: Minimum temperature.<br><br>T_{max}: Maximum temperature.<br><br>T_{opt}: Optimum temperature.<br><br>$\Delta H^{\ddagger}_{To}$: Change of enthalpy.<br><br>$\Delta S^{\ddagger}_{To}$: Change of entropy.<br><br>$\Delta C^{\ddagger}_{P}$: Change in heat capacity.

Table S3. Correlation (Pearson correlation coefficients) between soil properties and AOA to AOB ratios. Values in parentheses indicate significance values (*P)* determined by using t distribution.

Parameters	Organic farm			Inorganic farm			Organic + Inorganic farm		
	qAOA	qAOB	AOA/ AOB	qAOA	qAOB	AOA/ AOB	qAOA	qAOB	AOA/ AOB
**pH***	-0.28 (0.43)	-0.28 (0.43)	0.05 (0.88)	-0.36 (0.31)	-0.22 (0.54)	-0.02 (0.95)	-0.207 (0.38)	-0.14 (0.56)	-0.07 (0.78)
**TN**	0.42 (0.22)	0.58 (0.08)	-0.08 (0.84)	0.01 (0.98)	-0.13 (0.72)	0.01 (0.98)	0.18 (0.45)	0.22 (0.34)	0.064 (0.79)
**TOC**	-0.16 (0.66)	0.08 (0.82)	-0.45 (0.19)	-0.63 (0.047)	-0.43 (0.21)	-0.1 (0.79)	0.1 (0.67)	0.33 (0.15)	-0.30 (0.2)
**C/N ratio**	-0.41 (0.24)	-0.32 (0.37)	-0.31 (0.38)	-0.62 (0.057)	-0.37 (0.29)	-0.05 (0.89)	-0.01 (0.96)	0.16 (0.50)	0.27 (0.24)

*Soil to water ratio (1:2.5), qAOA: Abundances of ammonia oxidizing archaea, qAOB: Abundances of ammonia oxidizing bacteria, TN: Total nitrogen, TOC: Total organic carbon, C/N: TOC to TN ratio
